# Serum calcification propensity is independently associated with disease activity in systemic lupus erythematosus

**DOI:** 10.1371/journal.pone.0188695

**Published:** 2018-01-24

**Authors:** Suzan Dahdal, Vasilios Devetzis, George Chalikias, Dimitrios Tziakas, Carlo Chizzolini, Camillo Ribi, Marten Trendelenburg, Ute Eisenberger, Thomas Hauser, Andreas Pasch, Uyen Huynh-Do, Spyridon Arampatzis, on behalf of the Swiss Systemic Lupus Erythematosus Cohort Study Group

**Affiliations:** 1 Department of Nephrology and Hypertension lnselspital, Bern University Hospital, University of Bern, Bern, Switzerland; 2 Department of Cardiology, Medical School, Democritus University of Thrace, Alexandroupolis, Greece; 3 Division of Clinical Immunology and Allergy, Department of Internal Medicine Specialties, University Hospital and School of Medicine, Geneva, Switzerland; 4 Division of Clinical Immunology and Allergy, University Hospital Lausanne, Lausanne, Switzerland; 5 Division of Internal Medicine and Clinical Immunology Laboratory, Department of Biomedicine, University Hospital Basel, Basel, Switzerland; 6 Department of Nephrology, University Hospital Essen, University Duisburg-Essen, Duisburg, Germany; 7 Immunologie-Zentrum, Zurich, Switzerland; 8 Department of Biomedical Research, University of Bern, Bern, Switzerland; 9 Calciscon AG, Nidau, Switzerland; University of British Columbia, CANADA

## Abstract

**Background:**

Systemic lupus erythematosus (SLE) is associated with severe cardiovascular complications. The T_50_ score is a novel functional blood test quantifying calcification propensity in serum. High calcification propensity (or low T_50_) is a strong and independent determinant of all-cause mortality in various patient populations.

**Methods:**

A total of 168 patients with ≥ 4 American College of Rheumatology (ACR) diagnostic criteria from the Swiss Systemic lupus erythematosus Cohort Study (SSCS) were included in this analysis. Serum calcification propensity was assessed using time-resolved nephelometry.

**Results:**

The cohort mainly consisted of female (85%), middle-aged (43±14 years) Caucasians (77%). The major determinants of T_50_ levels included hemoglobin, serum creatinine and serum protein levels explaining 43% of the variation at baseline. Integrating disease activity (SELENA-SLEDAI) into this multivariate model revealed a significant association between disease activity and T_50_ levels. In a subgroup analysis considering only patients with active disease (SELENA-SLEDAI score ≥4) we found a negative association between T_50_ and SELENA-SLEDAI score at baseline (Spearman’s rho -0.233, P = 0.02).

**Conclusions:**

Disease activity and T_50_ are closely associated. Moreover, T_50_ levels identify a subgroup of SLE patients with ongoing systemic inflammation as mirrored by increased disease activity. T_50_ could be a promising biomarker reflecting SLE disease activity and might offer an earlier detection tool for high-risk patients.

## Introduction

Patients with systemic lupus erythematosus (SLE), a chronic inflammatory autoimmune disease, suffer from a dramatically increased cardiovascular morbidity and mortality compared to age- and gender matched individuals [1, 2]. Although the survival rates of patients with SLE has considerably improved over the last decades, a large proportion of this excess risk is attributable to causes, which can be only partially explained by traditional cardiovascular risk factors such as hypertension, hyperlipidemia, smoking, and obesity [3, 4]. Indeed, non-traditional cardiovascular risk factors reflecting inflammation, disease activity or oxidative stress may contribute to the increased cardiovascular risk in these patients [5–7].

Evidence from SLE animal models suggests that the degree of systemic inflammation correlates with the development of atherosclerosis, and vascular damage [8, 9]. Accelerated vascular micro-calcification, as a novel cellular, inflammation-driven pathway of arterial calcification, represents an intriguing potential mechanism linking accelerated vascular damage to inflammation and cardiovascular risk in SLE [10].

Fetuin-A is a potent regulator of extracellular matrix mineralization and the major serum-based inhibitor of calcium phosphate precipitation [11]. Dysregulation of Fetuin-A levels has been associated with increased systemic inflammation and pro-calcifying cytokine production [12]. Indeed, pro-inflammatory cytokines are considered important promoters of vascular smooth muscle cell osteochondrocytic transformation and mineralization [13]. Therefore, identification of circulating biomarkers for this process and systematic testing of their links with SLE disease activity and subsequent clinical cardiovascular events is of importance for advancing knowledge in this area of clinical research.

The T_50_ score, which represents the maturation time of calciprotein particles, is a novel biomarker, validated for the determination of serum calcification propensity [14]. The T_50_-Test is based on a nephelometric method allowing to quantify in-vitro the calcification inhibitory capacity of serum under predefined conditions of rising calcium and phosphate concentrations. A high calcification propensity (i.e. low T_50_) is associated with increased all-cause mortality and outperforms the predictive value of traditional cardiovascular risk factors concerning all-cause mortality in various patient populations with established chronic kidney disease (CKD) [15], on hemodialysis [16] and after kidney transplantation [17]. Furthermore, low T_50_ values were closely associated with progressive stiffening of the aorta [15].

However, to date, there are no clinical studies, which have investigated whether T_50_ values are associated with SLE disease activity. In this prospective study of SLE patients enrolled in the Swiss Systemic lupus erythematosus Cohort Study (SSCS), we hypothesize that T_50_ values, potentially as an indirect marker of systemic inflammation, are independently related to higher disease activity and adverse cardiovascular events.

## Materials and methods

### Patient population and study design

Our study population consists of SLE patients participating in the SSCS, a cooperative multicenter study across various clinical disciplines (clinical immunology, internal medicine, nephrology, rheumatology), in seven tertiary medical centers of Switzerland (Basel, Bern, Geneva, Lausanne, St. Gallen, Schaffhausen and Zurich) [18]. Characteristics and treatment modalities of the SSCS have been previously published [19]. SSCS has been approved by the ethics committees of all involved institutions (i.e. Ethikkommission Nordwest- und Zentralschweiz, Kantonale Ethikkommission Bern, Commission cantonale d'éthique de la recherche Genève, Commission cantonale d'éthique de la recherche sur l'être humain Vaud, Ethikkommission Ostschweiz, Ethikkommission des Kantons Zürich) and is in line with the declaration of Helsinki. All subjects gave their written informed consent in the context of the cohort study.

The present study was designed as a cross-sectional analysis of prospectively collected data between April 2007 and December 2013. Out of 180 patients (>18 years old) with available clinical, laboratory and baseline serum samples, 168 patients with ≥ 4 American College of Rheumatology (ACR) diagnostic criteria shown in S1 Table were included in the final analysis. Data regarding disease activity, biochemical characteristics, medication and cardiovascular risk factors were extracted from the cohort database. Target organ damage and prior cardiovascular events were captured at baseline. Serum samples were collected from each participant at cohort enrollment and were available for the calcification propensity (T_50_) assessment.

### Definitions

Definitions of clinical terms (cardiovascular events) and assessment tools (ACR criteria, SELENA-SLEDAI score, SLICC-DI) used throughout the manuscript are summarized in S2 Table. SLE was diagnosed using the 1997 revised classification ACR criteria. Disease activity was assessed by the “Safety of Estrogens in Lupus Erythematosus National Assessment—SLE Disease Activity Index” (SELENA-SLEDAI) (S2 Table) and a score ≥ 4 was considered as active disease in accordance with the definition used by the group of Yee C-S et al. [20] and previous analyses of the SSCS.

Organ damage was assessed by the Systemic Lupus International Collaborative Clinics/American College of Rheumatology (SLICC/ACR) Damage Index (SLICC/DI) summarized in S3 Table. A damage score of >1 was considered as severe damage while a score of 1 or lower was thought to reflect mild or no damage at all. Cardiovascular morbidity was defined using parameters exclusively recorded in the SLICC/DI-score summarized in S4 Table. Traditional as well as non-traditional cardiovascular risk factors were evaluated according to established definitions at each visit summarized in S5 Table. CKD, defined according to KDIGO 2012 criteria shown in S6 Table, was also captured as a non-traditional cardiovascular risk factor.

### Biochemical analyses

Serum samples were drawn from a peripheral vein in vacutainer tubes. After 30 to 60 minutes samples were centrifuged at 4000 rpm (corresponding to 2600 G) for 15 min at ambient temperature and the extracted serum was stored in aliquots and frozen at -80°C until further use. Serum calcification propensity was assessed using time-resolved nephelometry (BMG Labtech, Offenburg, Germany) according to an already described methodology [14]. All serum samples were measured under blinded conditions at the Department of Nephrology, Hypertension and Clinical Pharmacology, University Hospital Bern, Bern, Switzerland. Data were processed by calculating the precipitation time T_50_ from nonlinear regression curves. Samples were measured in triplicates. T50 is stable when samples were stored at -80°C throughout as has been demonstrated in previous studies [16]. Also no hemolytic sera were measured.

### Statistical analysis

Data are presented as absolute numbers with percentages for categorical data, as means ± standard deviation (SD) for continuous variables that were normally distributed and as medians with interquartile range (IQR) for non-normally distributed data. Normality was tested using the Kolmogorov-Smirnov test. A cross tabulation for comparison of all demographic, clinical and laboratory characteristics was performed across the 3 tertiles of serum T_50_. P-value was calculated by one-way ANOVA test for continuous variables with normal distribution, Kruskal-Wallis test for continuous variables with non-normal distribution and chi-squared test for categorical variables. Factors associated with baseline serum T_50_ were evaluated using linear regression models. Only variables which were significantly different between the 3 tertiles of serum T_50_ were analyzed. B-values were expressed per one SD increase in each continuous independent variable. In case of increased co-linearity between variables, the variable which conferred better to the R^2^ value was selected. After the conduction of multivariate analysis, forced models were designed forcing activity (SLEDAI) and damage (SLICC) indexes into the model. We also performed sensitivity analyses in various clinical, demographic and activity score subgroups of patients to examine the association between serum calcification propensity (T_50_ value) and several parameters. A p value < 0.05 was considered to indicate statistical significance with the exception of multiple comparisons, in which Bonferroni’s correction for multiple comparisons was applied and a p value < 0.016 (significant P value = P value / number of comparisons between groups = 0.05 / 3 = 0.016) was considered as significant; all tests were two-sided. The IBM SPSS Statistics 20.0 statistical software package (SPSS Inc, Chicago, Illinois, USA) was used for all calculations.

## Results

### Baseline characteristics

**Fig 1:**
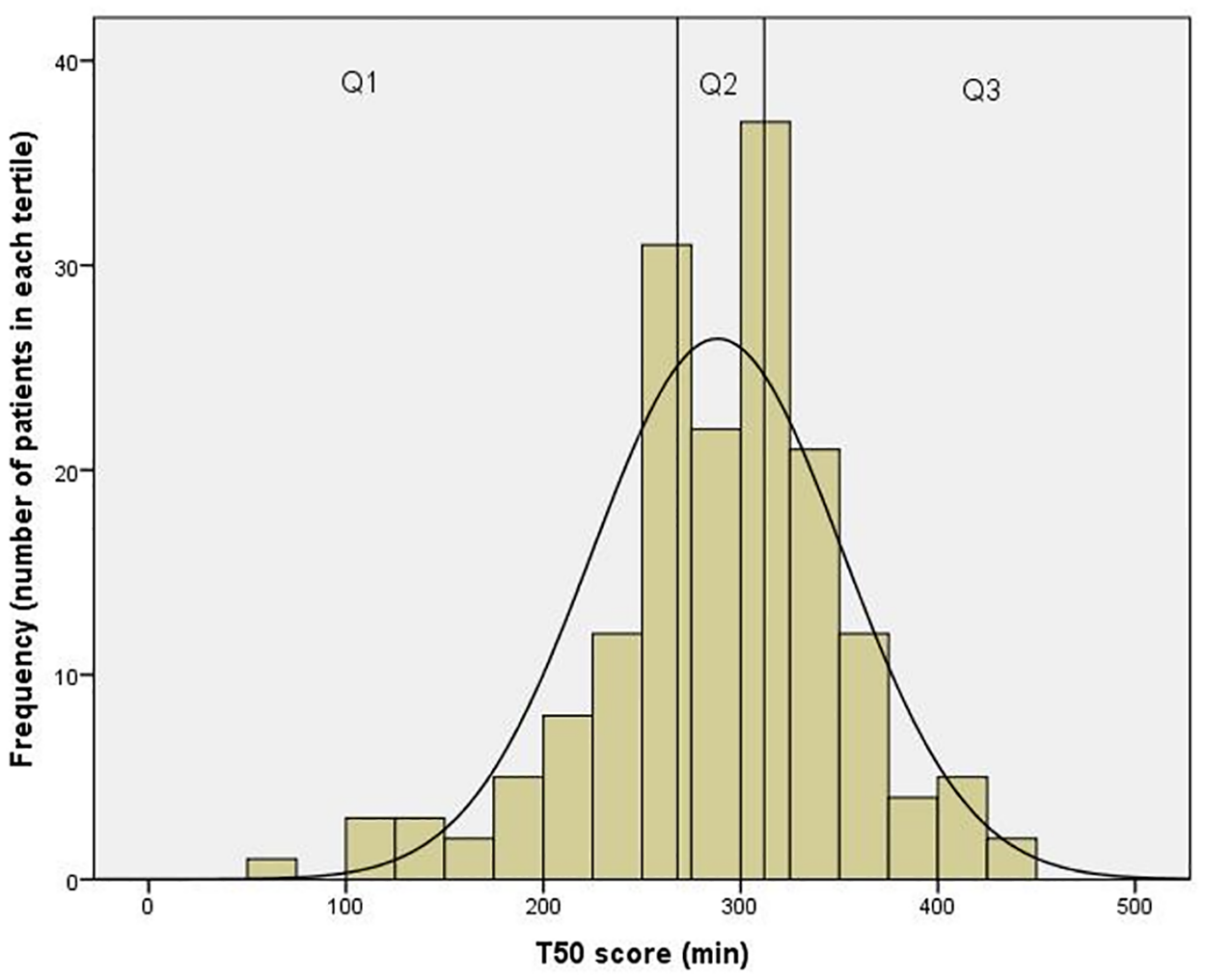
Distribution of serum calcification propensity score in SLE patients. Histogram of serum calcification propensity score (T_50_) distribution in SLE patients (Kurtosis 1.252, Skewness -0.623). Q1, Q2 and Q3 represent tertiles of T_50_ values.

Serum T_50_ measured at baseline displayed near-normal distribution (Fig 1). The baseline demographic, clinical and laboratory characteristics of all study individuals (n = 168) and across tertiles of serum T_50_ are presented in Table 1. The cohort consisted mainly of female (85%), middle-aged (43±14 years) outpatients of Caucasian origin (77%); 35% were overweight. The median (IQR) SLE-disease activity assessed by SELENA-SLEDAI score was 4 (7) reflecting mildly active lupus with a mean disease duration of 7 (11) years. The organ damage SLICC-DI score was low 0 (1) and the cohort exhibited a rather low occurrence of clinically documented atherosclerosis (15%) and a low prevalence of traditional cardiovascular risk factors. Participants had preserved renal function, with a GFR of 91 (±28.1) ml/min/1.73m^2^ and only 11% had CKD, as defined by the KDIGO criteria. A total of 13% had suffered a previous cardiovascular event at any time-point before the baseline measurement.

**Table 1:** Baseline characteristics at enrollment in the study cohort.

Characteristic	Overall (n = 168)	Low tertile (n = 54)	Intermediate tertile (n = 57)	High tertile (n = 57)	P value†
**Demographics**					
Age (years)	43±14	44±16	40±13	46±14	0.02
Female/ Male	142/26	43/11	48/9	51/6	0.35
Race n (%)					0.37
Caucasian	130 (77%)	37 (69%)	48 (84%)	45 (79%)	
African	15 (9%)	7 (13%)	3 (5%)	5 (9%)	
Asian	17 (10%)	8 (15%)	4 (7%)	5 (9%)	
Native American	3 (2%)	2 (4%)	1 (2%)	0	
Obese or overweight (BMI ≥25 kg/m^2^)	48 (35%)	19 (40%)	15 (32%)	14 (33%)	0.65
**SLE Characteristics**					
Disease duration (years)	7 (11)	6 (9)	6 (14)	8 (14)	0.67
Age at diagnosis (years)	31 (23)	33 (24)	28 (21)	33 (24)	0.05
ACR criteria (points)	5 (2)	5 (1)	5 (1)	5 (2)	0.52
SELENA-SLEDAI Score (points)	4 (7)	5 (13)	4 (8)	4 (7)	0.14
Proteinuria (>0.5g/24h) n,(%)	22 (13%)	14 (26%)	7 (12%)	1 (2%)	<0.001*
Hematuria n, (%)	26 (16%)	14 (26%)	5 (9%)	7 (12%)	0.008*
SLICC-DI (points)	0 (1)	0 (2)	0 (1)	0 (1)	0.41
**Atherosclerosis Risk Factors**					
Diabetes Mellitus n,(%)	8 (5%)	4 (7%)	1 (2%)	3 (5%)	0.36
Hypertension n,(%)	59 (35%)	23 (43%)	18 (32%)	18 (32%)	0.37
Current Smoking n,(%)	39 (23%)	11 (20%)	14 (25%)	14 (25%)	0.83
Dyslipidemia n,(%)	25 (15%)	9 (17%)	7 (12%)	9 (16%)	0.78
Chronic Kidney Disease n,(%) ‡	19 (11%)	8 (15%)	8 (14%)	3 (5%)	0.20
**Cardiovascular Events at Baseline**					
Coronary Artery Disease (%)	9 (5%)	4 (7%)	1 (2%)	4 (7%)	0.38
Cerebrovascular Disease (%)	11 (7%)	3 (6%)	5 (9%)	3 (5%)	0.60
Peripheral Arterial Disease (%)	3 (2%)	1 (2%)	0	2 (4%)	0.19
Atherosclerosis in all vascular beds (%)	21 (13%)	8 (15%)	6 (11%)	7 (13%)	0.81
**Cardiovascular History**					
Previous Myocardial Infarction (%)	4 (2%)	2 (4%)	1 (2%)	1 (2%)	0.75
Chronic Heart Failure (%)	2 (1%)	1 (2%)	0	1 (2%)	0.56
DVT / PE (%)	11 (7%)	6 (13%)	4 (7%)	1 (2%)	0.11
**Laboratory Values**					
Erythrocyte sedimentation rate (1st hour)	15 (28)	22 (47)	12 (21)	17 (27)	0.09
Hemoglobin (g/l)	128 (25)	124 (28)	129 (24)	129 (19)	0.008*
Serum creatinine (umol/l)	68 (22)	75 (35)	67 (19)	64 (21)	0.012*
GFR (ml/min/1.73m2)	91 (28)	91 (48)	95 (35)	99 (31)	0.29
Serum protein (g/l)	72 (8)	69 (13)	73 (9)	73 (8)	0.004*
Serum albumin (g/l)	38 (6)	35 (10)	39 (6)	40 (8)	<0.001*
Complement C3 (mg/dl)	0.84 (0.43)	0.72 (0.39)	0.87 (0.42)	0.94 (0.35)	0.016*
Complement C4 (mg/dl)	0.14 (0.1)	0.13 (0.09)	0.16 (0.09)	0.14 (0.1)	0.19
Proteinuria (24h collection, g/l)	0.15 (0.90)	0.65 (1.5)	0.15 (2.2)	0.1 (0.18)	0.40
**Medications**					
Systemic corticosteroids n,(%)	88 (52%)	34 (63%)	26 (46%)	28 (49%)	0.15
Immunosuppressants n,(%)	61 (36%)	24 (44%)	22 (39%)	15 (26%)	0.12
Other immunomodulators n,(%)	3 (2%)	1 (2%)	2 (4%)	0	0.24
NSAIDs n,(%)	24 (14%)	8 (15%)	6 (11%)	10 (18%)	0.82

Values are presented as mean values ± standard deviation or median values with (interquartile range) for continuous variables or as percentages for categorical variables. BMI, body mass index; SLE, systemic lupus erythematosus; ACR, American College of Rheumatology; SELENA-SLEDAI, Safety of Estrogen in Lupus Erythematosus National Assessment–Systemic Lupus Erythematosus Disease Activity Index; SLICC-DI, Systemic Lupus International Collaborative Clinics- Damage Index; DVT Deep Vein thrombosis; PE, Pulmonary Embolism; GFR, glomerular filtration rate; NSAIDs, non-steroid anti-inflammatory drugs.

* P value was considered as significant <0.016 for correction for multiple comparisons

^†^ P value was calculated by one-way ANOVA test for continuous variables with normal distribution, Kruskal-Wallis test for continuous variables with non-normal distribution and chi-squared test for categorical variables

^‡^ CKD defined as eGFR < 60 m/min

Dividing the cohort in tertiles of T_50_ resulted in three groups of patients with similar characteristics with regards to demographics, medication and main clinical parameters (Table 1). Concerning laboratory parameters, descending T_50_ tertiles (i.e, reflecting increasing serum calcification propensity) were associated with increasing prevalence of proteinuria (on 24-hours urine collection) and hematuria, and with decreasing levels of hemoglobin, serum protein and albumin, serum creatinine and C3 complement. Systemic corticosteroids were the most common treatment regimen in our cohort.

### Determinants of serum calcification propensity score (T_50_)

Univariate linear modeling showed that only hemoglobin, serum protein, albumin, creatinine and C3 complement levels were significantly associated with T_50_ levels (Table 2). After multivariate modeling, serum T_50_ levels remained associated with hemoglobin, serum creatinine and serum protein levels, explaining 43% of the variation in T_50_ at baseline. Forcing the disease activity marker SELENA-SLEDAI score into the model revealed a significant inverse association between disease activity and T_50_ levels. Implementation of the organ damage marker (SLICC-DI score) into the model showed a non-significant association between organ damage and T_50_ levels (data not shown).

**Table 2:** Determinants of serum calcium propensity score (T_50_) in univariate, multivariate linear regression analyses and in forced model using SELENA-SLEDAI score.

		T_50_					
		Univariate model		Multivariate model		Forced model	
Variable ^a^	SD increment	β value ^b^ (95%CI)	P value	β value ^b^ (95%CI)	P value	β value ^b^ (95%CI)	P value
Proteinuria (>0.5g/24h)	n/a	0.1 (-0.2 to 0.4)	0.58	-	-	-	-
Hematuria	n/a	0.4 (-0.1 to 0.8)	0.08	-	-	-	-
Hemoglobin (g/l)	17.8	37 (22 to 51)	<0.001	26 (3 to 48)	0.02	-24 (-42 to -0.7)	0.007
Serum Creatinine (μmol/l)	35.3	-31 (-46 to -17)	<0.001	-18 (-36 to -0.1)	0.04	37 (14 to 61)	0.002
Serum Protein (g/l)	7.9	55 (37 to 73)	<0.001	42 (18 to 65)	0.001	- ^c^	- ^c^
Serum Albumin (g/l)	7.2	57 (41 to 74)	<0.001	- ^c^	- ^c^	-	-
Complement C3 (mg/dl)	0.28	24 (6 to 42)	0.01	-	-	-	-
SELENA-SLEDAI score (points)	8					-70 (-118 to -21)	0.006

^a^ Only baseline variables that were different between the 3 tertiles of serum T_50_ (P<0.016) were analyzed.

^b^ Per 1 SD increase in each continuous independent variable

^c^ Due to increased co linearity between serum protein and serum albumin levels, the variable which conferred better to the R^2^ value was selected (i.e. serum protein levels)

All continuous variables were divided by their corresponding standard deviation in order to achieve normal distribution. SELENA-SLEDAI; Safety of Estrogen in Lupus Erythematosus National Assessment–Systemic Lupus Erythematosus Disease Activity Index

### Serum calcification propensity score (T_50_) and cardiovascular events

At baseline 21 patients (13%) had clinically documented atherosclerosis in various vascular beds. Table 3 depicts univariate association of several risk factors of atherosclerosis and of T_50_ with total cardiovascular events. T_50_ levels tended to be marginally associated with presence of adverse cardiovascular events (OR 2.2 95% CI 0.9–5.8 per 1 SD change; p = 0.099). Of interest, T_50_ levels remained–albeit marginally significant- an independent predictor (OR 2 95% CI 0.9–4.4 per 1 SD change; p = 0.074) for prevalent cardiovascular disease in a multivariate regression model (Table 3) that incorporated all significant confounders (p = 0.100).

**Table 3:** Univariate and multivariate association of known predictors of atherosclerotic disease and serum calcification propensity score (T_50_) at baseline with total cardiovascular events.

Variable ^a^	Total Cardiovascular Events			
	Univariate		Multivariate	
	ORs^b^ (95%CI)	P value	ORs^b^ (95%CI)	P value
Age (years)	2 (1.3–3.1)	0.003		
Obese or overweight (BMI ≥25 kg/m^2^)	1.6 (0.5–4.5)	0.41		
Diabetes mellitus	4.6 (1–21)	0.04		
Dyslipidemia	7.8 (2.9–21.5)	<0.001	8.9 (2–39.5)	0.004
Hypertension	4.6 (1.7–12)	0.002		
Smoking	1.8 (0.7–4.7)	0.26		
Chronic Kidney Disease	2.9 (0.9–9.1)	0.06		
Disease duration (months)	1.6 (1.1–2.4)	0.01		
SELENA-SLEDAI (points)	1.8 (0.7–4.5)	0.19		
SLICC-DI score (points)	4.7 (2.7–8.3)	<0.001	5.3 (2.5–11.3)	<0.001
GFR (ml/min/1.73m^2^)	0.9 (0.5–1.4)	0.51		
Systemic corticosteroids	3.5 (1.2–10)	0.02		
Lipid Lowering Drugs	5 (1.7–14.7)	0.003		
ACE-inhibitors	0.2 (0.1–0.6)	0.005		
AT-receptor blockers	0.6 (0.1–3)	0.54		
B-blockers	0.2 (0.1–0.6)	0.005		
Antithrombotics^c^	0.1 (0.1–0.2)	<0.001	0.1 (0.1–0.5)	0.004
Serum T_50_	2.2 (0.9–5.8)	0.09	2 (0.9–4.4)	0.07

^a^ In the multivariate model all baseline variable with P<0.100 were included.

^b^ Per 1 SD increase in each continuous independent variable (age, 14 years; disease duration, 9.2 months; SELENA-SLEDAI score, 8 points, SLICC-DI score, 2 points; serum/plasma creatinine, 35.3 umol/L; GFR, 27.8 ml/min/1.73m^2^; Total Cholesterol, 1.26 mmol/L; LDL cholesterol, 1.02 mmol/L; HDL cholesterol, 0.49 mmol/L; Triglycerides, 1.29 mmol/L; T_50_, 63 min)

^c^ Antithrombotic therapy included antiplatelet, oral anticoagulant, or low weight molecular weight heparin

BMI, Body Mass Index; SELENA-SLEDAI, Safety of Estrogen in Lupus Erythematosus National Assessment; Systemic Lupus Erythematosus Disease Activity Index; SLICC-DI, Systemic Lupus International Collaborative Clinics- Damage Index; GFR, glomerular filtration rate; ACE, Angiotensin Converting Enzyme; AT, angiotensin.

### Sensitivity analysis: T_50_ and disease activity

Given the association between T_50_ and disease activity, a subgroup-analysis was performed. Based on clinically definitions SELENA-SLEDAI ≥4 was considered active SLE, whereas a score <4 was considered inactive disease [20]. T_50_ showed no difference (p = 0.104) between the subgroup of SLE patients with inactive (T_50_ median (IQR) levels; 303 (66) min.) or active disease (T_50_ median (IQR); 291 (80) min.). Furthermore, distribution of active or inactive disease was similar across the tertiles of T_50_ (low tertile: 33% inactive vs 67% active; intermediate tertile: 44% inactive vs. 56% active; high tertile: 47% inactive vs. 53% active; χ^2^-test, P = 0.298). However, when considering only patients with active disease (SELENA-SLEDAI score ≥4; n = 98) we found a significant negative association between T_50_ and SELENA-SLEDAI score at baseline (Spearman’s rho -0.233, P = 0.021). In contrast, in SLE patients with inactive disease (SELENA-SLEDAI score <4; n = 70), no significant association between baseline T_50_ and SELENA-SLEDAI score was found (Spearman’s rho -0.051, P = 0.675) and summarized in S1 Fig.

In order to explore the impact of mild disease activity, we performed a subgroup-analysis considering SELENA-SLEDAI >0 as active and a score of 0 as inactive SLE disease. T_50_ showed no difference (p = 0.414) between the subgroup of SLE patients with inactive (n = 37; T_50_ median (IQR) levels; 301 (65) min.) or active disease (n = 131; T_50_ median (IQR); 292 (69) min. However, when considering only patients with active disease (SELENA-SLEDAI score >0; n = 131) we found a pronounced negative association between T50 and SELENA-SLEDAI score at baseline (Spearman’s rho -0.219, P = 0.012).

### Sensitivity analysis: Clinical and demographic subgroups

Sensitivity analysis of demographics revealed that the observed inverse correlation between serum calcification propensity score (T_50_) and disease activity (SELENA-SLEDAI score) was associated with male sex (Spearman’s rho -0.174; p = 0.039), obesity (BMI ≥ 25 kg/m2; Spearman’s rho -0.344; p = 0.017), GFR < 60ml/min/1.73 m^2^ (Spearman’s rho -0.521; p = 0.009), diabetes mellitus (Spearman’s rho -0.854; p = 0.007), hypertension (Spearman’s rho -0.345; p = 0.008), non-smoking status (Spearman’s rho -0.148; p = 0.017), and the absence of dyslipidemia (Spearman’s rho -0.165; p = 0.048) (Table 4).

**Table 4:** Interaction effects of various clinical and demographic variables on the observed association between serum calcification propensity score (T_50_) and disease activity (SELENA-SLEDAI).

Variable	Spearman’srho	P value	Fisher’s z-test	P value for Interaction
Male	-0.174	0.03	0.335	0.73
Female	-0.246	0.22		
Age <70 years	-0.153	0.05	1.217	0.22
Age ≥70 years	-0.609	0.10		
Low GFR <60 min/min/1.73m2	-0.521	0.009	1.962	0.04
High GFR ≥ 60 ml/min/1.73m2	-0.115	0.18		
No Albuminuria (<0.3 g/l)	-0.015	0.95	0.631	0.52
Albuminuria (≥0.3 g/l)	-0.299	0.47		
Low LDL cholesterol (<100 mg/dl)	-0.097	0.62	0.679	0.49
High LDL cholesterol (≥100 mg/dl)	-0.298	0.19		
Low SBP (<135mmHg)	-0.123	0.24	1.088	0.27
High SBP (≥135mmHg)	-0.379	0.08		
No Diabetes Mellitus	-0.143	0.07	2.480	0.01
Diabetes Mellitus	-0.854	0.007		
No Dyslipidemia	-0.165	0.04	0.332	0.73
Dyslipidemia	-0.238	0.25		
No Hypertension	-0.061	0.53	1.808	0.07
Hypertension	-0.345	0.008		
No Smoking	-0.210	0.01	0.474	0.63
Smoking	-0.123	0.45		
No Chronic Kidney Disease	-0.148	0.07	0.865	0.38
Chronic Kidney Disease	-0.360	0.13		
No obesity (BMI < 25 Kg/m^2^)	-0.090	0.40	1.456	0.14
Obesity (BMI ≥ 25 Kg/m^2^)	-0.344	0.01		

SELENA-SLEDAI, Safety of Estrogen in Lupus Erythematosus National Assessment–Systemic Lupus Erythematosus Disease Activity Index; GFR, glomerular filtration rate; LDL, Low Density Lipoprotein; SBP, Systolic Blood Pressure; BMI, Body Mass Index. Subsequent analysis for interaction showed that GFR <60 ml/min/1.73m^2^, diabetes mellitus, and hypertension significantly influenced the inverse association between serum calcification propensity T_50_ score and disease activity (Table 4). Multivariate analysis in a model of all possible interaction terms (diabetes mellitus, hypertension, GFR levels) revealed that serum calcification propensity score (T_50_) continued to be inversely associated with disease activity as described by SELENA-SLEDAI score (beta value -86 95% CI -126 to -46 per 1 SD change (8 points) of SELENA-SLEDAI score; p<0.001).

## Discussion

Cardiovascular disease is one of the major causes of morbidity and mortality in SLE. The T_50_-value is a novel integrated functional measure of calcification propensity in human blood (serum), which may mechanistically link cardiovascular risk and vascular damage with SLE disease activity. The present study explored for the first time a potential association between T_50_ and SLE activity in a cohort of relatively young SLE patients. As our main finding we demonstrated that low serum T_50_ was associated with SELENA-SLEDAI score, in particular in patients with active lupus disease.

T_50_ quantifies the overall calcification-propensity, by timing the spontaneous transformation of spherical primary calciprotein particles (CPPs) to spindle-shaped, stabilized secondary CPPs, which contain crystalline calcium phosphate [14]. In our study, several parameters, such as low hemoglobin, increased serum creatinine and low serum protein levels, were significantly associated with T_50_ levels. All these factors reflecting disease activity and indirectly systemic inflammation, explain after multivariate modeling 43% of the variation of T_50_ level at baseline. Analogously, Smith et al found in their cohort of patients with mild to moderate CKD an association between lower serum T_50_ values and higher concentrations of the inflammatory markers hsCRP and TNF-α [15]. Experimental models demonstrate that secondary CPP provoke proinflammatory responses in murine macrophages [21] and also in vascular smooth muscle cells by stimulating the release of TNF-α [22], relating the correlation of T_50_ with inflammation. After integrating the disease activity marker SELENA-SLEDAI score into the model, a significant inverse association between disease activity and T_50_ levels was revealed. Of interest, this significant inverse association can still be found considering mild disease activity. These observations support our hypothesis that humoral mineralization imbalances may play a pathogenic role in premature cardiovascular in SLE.

Although there were a low incidence of atherosclerosis and previous cardiovascular events in our cohort, T_50_ levels were a near-significant independent predictor for cardiovascular disease. In our study, traditional cardiovascular risk factors (i.e. age, sex, dyslipidemia or smoking) were not identified as determinants of T_50_ in regression analysis. Our results open the possibility that calcification propensity may represent a non-traditional cv-risk factors important not only for renal patients but also for patients suffering from chronic inflammatory diseases, which are of note also associated with increased levels of endogenous calciprotein particles [23]. Data from animal models suggest that the vascular endothelium in SLE is more prone to inflammation [24]. Similarly, disease activity, longer duration of disease, higher damage-index score and less aggressive immunosuppressive therapy were shown to be important determinants of accelerated atherosclerosis in SLE [1, 6, 7, 25–27], suggesting a potential atherogenic effect of inflammation in SLE. Nevertheless no significant correlation between inflammatory markers—such as CRP, interleukin-6 or tumor necrosis factor—and accelerated atherosclerosis in SLE were found [1, 2, 7, 25, 26]. The factors responsible for accelerated atherosclerosis in SLE patients seem to be more complex and difficult to be determined by the measurement of a single molecule. This is not entirely surprising since the mechanisms driving mineralization are multifactorial and dependent on a balance between calcification inhibitors and promoters. Given that T_50_ is a functional test of the all over capacity of calcification, different factors influencing the process of calcification are taken into account, whether these factors inhibit or promote calcification or whether these factors are known or not yet known. The T_50_ value may therefore provide an important tool linking inflammation, disease activity and accelerated atherosclerosis to the pronounced cardiovascular morbidity and mortality of SLE patients.

Our results support the proposed interplay of non-traditional and lupus-specific risk factors in the development of premature atherosclerosis driven by impaired “bio-mineralization”. Cardiovascular events and vascular calcification in multiple vascular beds are more prevalent in SLE that in age-matched subjects of the general population [4, 5]. In previous reports the risk of vascular calcification in patients with SLE was 33.6 fold higher than control subjects after adjusting for age and sex. Also in a cohort of 139 young lupus patients (93% females), which was screened for coronary artery calcifications Juanita Romero-Dıaz and colleagues found an association of coronary artery calcifications with increased disease activity along the course of lupus [28]. In accordance with previous findings, in our study cohort coronary artery calcification was already present despite the patients’ young age. Several studies suggest that treatment of disease activity with antimalarial drugs reduces atherosclerosis and thrombosis in SLE patients [1, 29–31]. The results of our study are in accordance with previous findings indicating that the T_50_ value may represent a composite of non-traditional cardiovascular risk factors and has already shown discriminatory ability concerning cardiovascular events in various patient cohorts. Increased serum calcification propensity (i.e., lower serum T_50_) was a potent predictor and risk factor of all-cause and cardiovascular mortality in long-term renal transplanted patients, which substantially improved mortality prognostication [17]. Also in a prospective cohort of 184 patients with stages 3 and 4 CKD and a follow-up time over 5 years, the lowest T_50_ tertile had in the fully adjusted multivariate analysis more than twice elevated mortality risk in comparison to patients in the highest T_50_ tertile [15].

Numerous established laboratory tests such as vitamin D receptors, interferon signature, urinary interleukin (IL)-6, and complement activation markers, assessing disease activity have been reported. However, their utility is limited because they are mainly experimental and not accessible for routine clinical care. Additionally they do not assess the risk for accelerated atherosclerosis due to disease activity. In our cohort we observed a significant association of higher serum calcification propensity at baseline with disease activity. Therefore, calcification propensity might bear the potential to routinely identify SLE patients with potential higher burden of cardiovascular disease and elevated risk due to non-traditional cardiovascular risk factors.

Our demonstration that T_50_ is closely associated with established non-traditional risk factors and reflects the patients’ previous cardiovascular morbidity is an important step towards a better understanding of the pathophysiological mechanisms involved in cardiovascular events in patients with SLE.

Our study is subject to limitations inherent to the observational nature of a registry dataset collected prospectively. The distribution curve of SELENA-SLEDAI score measurements at baseline shows a slight deviation to the right suggesting that the activity score is measured within a population with average low disease activity, minor organ damage and low prevalence of cardiovascular risk factors, limiting the generalizability of our findings to patients with more severe SLE manifestations and comorbidities. The study size did not have adequate statistical power to prove prognosis associations. Nevertheless, our findings provide important hints for an association with CV prognosis. Most of our subjects were Caucasian women, which limits the application of our findings to men and other ethnic backgrounds. Although a multitude of demographic, clinical, and disease related variables were adjusted in our multivariate analyses, as with all observational studies, it is possible that unmeasured confounders may have influenced our results.

In conclusion, this is the first study to demonstrate an association between lower T_50_ and disease activity. Moreover, T_50_ levels identify a subgroup of SLE patients with ongoing systemic inflammation as mirrored by increased disease activity. Notably, all of these relationships persisted even after adjustment for other biomarkers and comorbidities. Based on these findings, we postulate that T_50_ could be a promising biomarker reflecting SLE disease activity and might offer an earlier detection tool for high-risk patients with non-traditional cardiovascular risk factors. Additional studies are needed to corroborate these findings and guide the implementation of T_50_ in cardiovascular preventive strategies and aggressive management in selected SLE patients.

## Supporting information

S1 Table 1997 Update of the 1982 American College of Rheumatology revised criteria for classification of systemic lupus erythematosus. (DOC)

S2 Table SELENA-SLEDAI (systemic lupus erythematosus Disease Activity Index). (DOC)

S3 Table Systemic Lupus International Collaborating Clinics/American College of Pneumatology Damage Index for systemic lupus erythematosus (SLICC/ACR-DI). (DOC)

S4 Table Definitions of clinical composites of cardiovascular morbidity. (DOC)

S5 Table Study-specific definition of cardiovascular risk factors. (DOC)

S6 Table KDIGO 2012 criteria for the classification of chronic kidney disease (CKD). (DOC)

S1 Fig Association between T50 and SELENA-SLEDAI score at baseline in disease activity. (DOC)
