# Supplementary material for: Serum calcification propensity is independently associated with disease activity in systemic lupus erythematosus
Source: PLoS One. 2018 Jan 24;13(1):e0188695. doi: 10.1371/journal.pone.0188695 (PMC5783342; doi:10.1371/journal.pone.0188695)
Supplement: S1 Table — (DOC) [file pone.0188695.s001.doc]

**S1 Table. 1997 Update of the 1982 American College of Rheumatology Revised Criteria for Classification of Systemic Lupus Erythematosus (1)**

| **Criterion** | **Definition** |
| --- | --- |
| 1. Malar rash | Fixed Erythema, flat or raised, over the malar eminences, tending to spare the nasolabial folds |
| 1. Discoid rash | Erythematous raised patches with adherent keratotic scaling and follicular plugging; Atrophic scaring may occur in older lesions |
| 1. Photosensitivity | Skin rash as a result of unusual reaction to sunlight, by patients history or physician observation |
| 1. Oral ulcers | Oral or nasopharyngeal ulceration, usually painless, observed by physician |
| 1. Nonerosive arthritis | Involving 2 or more peripheral joints, characterized by tenderness, swelling or effusion |
| 1. Pleuritis or Pericarditis | Pleuritis-convincing history of pleuritic pain or rubbing heard by a physician or evidence of pleural effusion OR  Pericarditis-documented by electrocardiogram or rub or evidence of pericardial effusion |
| 1. Renal disorder | Persistent Proteinuria > 0.5 g per day or > than 3+ if quantitation not performed OR  Cellular casts-may be red cell, hemoglobin, granular, tubular, or mixed |
| 1. Neurologic disorder | Seizures in the absence of offending drugs or known metabolic derangements; e.g., uremia, ketoacidosis, or electrolyte imbalance OR  Psychosis-in the absence of offending drugs or known metabolic derangements, e.g., uremia, ketoacidosis, or electrolyte imbalance |
| 1. Hematologic disorders | Hemolytic anemia-with reticulocytosis OR  Leucopenia - < 4,000/mm3 on ≥ 2 occasions OR  Lymphopenia - < 1,500/ mm3 on ≥ 2 occasions OR  Thrombocytopenia - < 100,000/mm3 in the absence of offending drugs |
| 1. Immunologic disorders | Anti-DNA: antibody to native DNA in abnormal titer OR  Anti-Sm: presence of antibody to SM nuclear antigen OR  Positive findings of antiphospholipid antibodies on:  1. an abnormal serum level of IgG or IgM anticardiolipin antibodies  2. a positive test result for lupus anticoagulant using a standard method, or  3. a false-positive test result for at least 6 months confirmed by Treponema pallidum immobilization or fluorescent treponemal antibody absorption test |
| 1. Positive Antinuclear Antibody | An abnormal titer of antinuclear antibody by immunofluorence or an equivalent assay at any point in time in the absence of drugs |

Patients have to fulfill ≥ 3 criteria to be included in the SSCS

Patients had to fulfill ≥ 4 criteria to be included in the present study

# Reference

1. Hochberg MC: Updating the American College of Rheumatology revised criteria for the classification of systemic lupus erythematosus. *Arthritis Rheum,* 40**:** 1725, 1997
