# Supplementary material for: Serum calcification propensity is independently associated with disease activity in systemic lupus erythematosus
Source: PLoS One. 2018 Jan 24;13(1):e0188695. doi: 10.1371/journal.pone.0188695 (PMC5783342; doi:10.1371/journal.pone.0188695)
Supplement: S2 Table — (DOC) [file pone.0188695.s002.doc]

**S2 Table. SELENA-SLEDAI (Systemic Lupus Erythematosus Disease Activity Index)**

| **Weight** | **Present** | **Descriptor** | **Definition** |
| --- | --- | --- | --- |
| 8 | **□** | Seizure | Recent onset (last 10 days). Exclude metabolic, infectious or drug cause, or seizure due to past irreversible CNS damage |
| 8 | □ | Psychosis | Altered ability to function in normal activity due to severe disturbance in the perception of  reality. Include hallucinations; incoherence; marked loose associations; impoverished  thought content; marked illogical thinking; bizarre, disorganized or catatonic behavior.  Exclude uremia and drug causes. |
| 8 | **□** | Organic brain syndrome | Altered mental function with impaired orientation, memory or other intellectual function, with  rapid onset and fluctuating clinical features. Include clouding of consciousness with  reduced capacity to focus, and inability to sustain attention to environment, plus at least 2 of  the following: perceptual disturbance, incoherent speech, insomnia or daytime drowsiness,  or increased or decreased psychomotor activity. Exclude metabolic, infectious or drug  causes. |
| 8 | **□** | Visual disturbance | Retinal and eye changes of SLE. Include cytoid bodies, retinal hemorrhages, serous exudate or  hemorrhages in the choroid, optic neuritis, scleritis or episcleritis. Exclude hypertension,  infection or drug causes. |
| 8 | **□** | Cranial nerve disorder | New onset of sensory or motor neuropathy involving cranial nerves. Include vertigo due to  lupus. |
| 8 | **□** | Lupus headache | Severe persistent headache: may be migrainous, but must be nonresponsive to narcotic analgesia. |
| 8 | **□** | CVA | New onset of cerebrovascular accident(s). Exclude arteriosclerosis or hypertensive causes. |
| 8 | **□** | Vasculitis | Ulceration, gangrene, tender finger nodules, periungual infarction, splinter hemorrhages, or biopsy or angiogram proof of vasculitis. |
| 4 | **□** | Arthritis | More than 2 joints with pain and signs of inflammation (i.e., tenderness, swelling or effusion). |
| 4 | **□** | Myositis | Proximal muscle aching/weakness, associated with elevated creatine phosphokinase/aldolase or  electromyogram changes or a biopsy showing myositis.. |
| 4 | **□** | Urinary casts | Heme-granular or red blood cell casts. |
| 4 | **□** | Hematuria | >5 red blood cells/high power field. Exclude stone, infection or other cause. |
| 4 | **□** | Proteinuria | New onset or recent increase of more than 0.5 gm/24 hours. |
| 4 | **□** | Pyuria | >5 white blood cells/high power field. Exclude infection. |
| 2 | **□** | Rash | Ongoing inflammatory lupus rash. |
| 2 | **□** | Alopecia | Ongoing abnormal, patchy or diffuse loss of hair due to active lupus. |
| 2 | **□** | Mucosal ulcers | Ongoing oral or nasal ulcerations due to active lupus. |
| 2 | **□** | Pleurisy | Classic and severe pleuritic chest pain or pleural rub or effusion or new pleural thickening due to  lupus. |
| 2 | **□** | Pericarditis | Classic and severe pericardial pain or rub or effusion, or electrocardiogram confirmation. |
| 2 | **□** | Low complement | Decrease in CH50, C3 or C4 below the lower limit of normal for testing laboratory. |
| 2 | **□** | Increased DNA binding | >25% binding by Farr assay or above normal range for testing laboratory. |
| 1 | **□** | Fever | >38oC. Exclude infectious cause. |
| 1 | **□** | Thrombocytopenia | <100,000 platelets/mm3. |
| 1 | **□** | Leukopenia | <3,000 white blood cells/mm3. Exclude drug causes. |
| **______ TOTAL SCORE** (Sum of weights next to descriptors marked present) | | | |

Box should be checked if descriptor is present at the time of visit or in the preceding 10 days.
