# Supplementary material for: Serum calcification propensity is independently associated with disease activity in systemic lupus erythematosus
Source: PLoS One. 2018 Jan 24;13(1):e0188695. doi: 10.1371/journal.pone.0188695 (PMC5783342; doi:10.1371/journal.pone.0188695)
Supplement: S3 Table — (DOC) [file pone.0188695.s003.doc]

**S3 Table. Systemic Lupus International Collaborating Clinics/American College of Pneumatology Damage Index for Systemic Lupus Erythematosus (SLICC/ACR-DI) (1)**

| Item | Score |  |
| --- | --- | --- |
| OCULAR (either eye, by clinical assessment) |  |  |
| Any cataract ever | 1 |  |
| Retinal change or optic atrophy | 1 |  |
| NEUROPSYCHIATRIC |  |  |
| Cognitive impairment (e.g. memory deficit, difficulty with calculation, poor concentration, difficulty in spoken or written language, impaired performance level) OR | 1 |  |
| Major psychosis |  |  |
| Seizures requiring therapy for **)**6 months | 1(2) |  |
| Cerebrovascular accident ever (score 2 if >1) | 1 |  |
| Cranial or peripheral neuropathy (excluding optic) | 1 |  |
| Transverse myelitis | 1 |  |
| RENAL |  |  |
| Estimated or measured glomerular filtration rate (GFR) < 50 % | 1 |  |
| Proteinuria > 3.5gr/24h OR |  |  |
| End Stage Renal Disease (regardless of dialysis or transplantation) | 1 |  |
| PULMONARY |  |  |
| Pulmonary hypertension (right ventricular prominence, or loud P2) | 1 |  |
| Pulmonary fibrosis (clinical and radiograph) | 1 |  |
| Shrinking lung (radiograph) | 1 |  |
| Pleural fibrosis (radiograph) | 1 |  |
| Pulmonary infarction (radiograph or resection not for malignancy) | 1 |  |
| CARDIOVASCULAR |  |  |
| Angina or coronary artery bypass | 1 |  |
| Myocardial infarction ever (score 2 if >1) | 1(2) |  |
| Cardiomyopathy (ventricular dysfunction) | 1 |  |
| Valvular disease (diastolic murmur, or a systolic murmur > 35) | 1 |  |
| Pericarditis x 6 months or pericardiectomy | 1 |  |
| PERIPHERAL VASCULAR |  |  |
| Claudication x 6 months | 1 |  |
| Minor tissue loss (pulp space) | 1 |  |
| Significant tissue loss ever (e.g. loss of digit or limb, resection) (Score 2 if >1) | 1(2) |  |
| Venous thrombosis with swelling. ulceration. OR venous stasis | 1 |  |
| GASTROINTESTINAL |  |  |
| Infarction or resection of bowel (below duodenum), spleen, liver or gall bladder ever, for any cause (score 2 if > 1 site) | 1(2) |  |
| Mesenteric insufficiency | 1 |  |
| Chronic peritonitis | 1 |  |
| Stricture OR upper gastrointestinal tract surgery ever | 1 |  |
| Pancreatic insufficiency requiring enzyme replacement or with pseudocyst | 1 |  |
| MUSCULOSKELETAL |  |  |
| Muscle atrophy or weakness | 1 |  |
| Deforming or erosive arthritis (including reducible deformities, excluding avascular necrosis) | 1 |  |
| Osteoporosis with fracture or vertebral collapse (excluding avascular necrosis) | 1 |  |
| Avascular necrosis (Score 2 if > 1) | 1 |  |
| Osteomyelitis | 1 |  |
| Ruptured tendons | 1 |  |
| SKIN |  |  |
| Scarring chronic alopecia | 1 |  |
| Extensive scarring or panniculum other than scalp and pulp space | 1 |  |
| Skin ulceration (excluding thrombosis) for > 6 months | 1 |  |
| PREMATURE GONADAL FAILURE | 1 |  |
| DIABETES (regardless of treatment) | 1 |  |
| MALIGNANCY (exclude dysplasia) (Score 2 if > 1 site) | 1(2) |  |
| Damage is considered a nonreversible change, not related to active inflammation, occurring since onset of lupus, ascertained by clinical assessment and present for at least 6 months unless otherwise stated. | |  |
| Repeat episodes must occur at least 6 months apart to score 2. | |  |
| The same lesion cannot be scored twice | |  |
|  | | |

# Reference

1. Stoll T, Seifert B, Isenberg DA: SLICC/ACR Damage Index is valid, and renal and pulmonary organ scores are predictors of severe outcome in patients with systemic lupus erythematosus. *Br J Rheumatol,* 35**:** 248-254, 1996
