# Supplementary material for: Serum calcification propensity is independently associated with disease activity in systemic lupus erythematosus
Source: PLoS One. 2018 Jan 24;13(1):e0188695. doi: 10.1371/journal.pone.0188695 (PMC5783342; doi:10.1371/journal.pone.0188695)
Supplement: S4 Table — (DOC) [file pone.0188695.s004.doc]

**S4 Table. Definitions of clinical composites of cardiovascular morbidity**

| **Clinical Term** | **Definition criteria** (based on SLICC/ACR-DI) |
| --- | --- |
| Cardiac events | One (1) or more myocardial infarctions, previous coronary artery bypass grafting (CABG), previous percutaneous coronary intervention (PCI) or occurrence of typical angina |
| Cerebral events | One (1) or more cerebrovascular insults with reversible or irreversible functional deficit with a related structural damage |
| Peripheral vascular events | Claudication and/or minor or major (at least loss or resection of a digit) simple or multiple tissue loss |
