# Supplementary material for: Serum calcification propensity is independently associated with disease activity in systemic lupus erythematosus
Source: PLoS One. 2018 Jan 24;13(1):e0188695. doi: 10.1371/journal.pone.0188695 (PMC5783342; doi:10.1371/journal.pone.0188695)
Supplement: S5 Table — (DOC) [file pone.0188695.s005.doc]

**S5 Table. Study-specific definition of cardiovascular risk factors**

| **Clinical term** | **Definition** |
| --- | --- |
| **Traditional cardiovascular risk factors** | |
| Hypertension (HTN) | Defined according to the JNC 7 diagnostic criteria (1). Considered prevalent when being under treatment for HTN at the time of the initial assessment of metabolic comorbidity and/or as receiving antihypertensives at the time-point of each T_50_ measurement. |
| Diabetes mellitus (DM) | Defined as a positive medical history of DM. Considered prevalent either when being under treatment for DM at the time of the initial assessment of metabolic comorbidity and/or as receiving anti-diabetic drugs at the time-point of each T_50_ measurement. |
| Dyslipidemia | Defined as LDL cholesterol levels >160 mg/d and/or total cholesterol levels >240 mg/dl and/or HDL cholesterol levels <40 mg/dl and/or triglyceride levels >150 mg/dl. Considered prevalent when being were under treatment for hyperlipidemia at the time of the initial assessment of metabolic comorbidity (after inclusion in the cohort) and/or as receiving lipid-lowering drugs at the time-point of each T_50_ measurement. |
| Smoking status | Defined as have been a previous or as being a current smoker. |
| Body mass index | Defined as weight in kilograms divided by squared height in meters (kg/m^2^) |
| **Non-traditional cardiovascular risk factors** | |
| Chronic kidney disease (CKD) | Defined according to KDIGO 2012 criteria. Considered prevalent when being present at the time of the initial assessment of metabolic comorbidity or when being under chronic dialysis therapy or as being transplanted. |
| Systemic lupus disease activity | According to the Systemic Lupus Erythematosus Disease Activity Index (SELENA-SLEDAI) |
| Organ damage | According to the Systemic Lupus International Collaborating Clinics/American College of Pneumatology Damage Index for Systemic Lupus Erythematosus (SLICC/ACR-DI) |

# Reference

1. Chobanian AV, Bakris GL, Black HR, Cushman WC, Green LA, Izzo JL, Jr., Jones DW, Materson BJ, Oparil S, Wright JT, Jr., Roccella EJ: The Seventh Report of the Joint National Committee on Prevention, Detection, Evaluation, and Treatment of High Blood Pressure: the JNC 7 report*. JAM*A, 2**89**: 2560-2572, 2003
