# Supplementary material for: Serum calcification propensity is independently associated with disease activity in systemic lupus erythematosus
Source: PLoS One. 2018 Jan 24;13(1):e0188695. doi: 10.1371/journal.pone.0188695 (PMC5783342; doi:10.1371/journal.pone.0188695)
Supplement: S6 Table — (DOC) [file pone.0188695.s006.doc]

**S6 Table. KDIGO 2012 criteria for the classification of chronic kidney disease (CKD) (1)**

| **Criteria for CKD (either of the following present for >3 months)** | | | | | | | |
| --- | --- | --- | --- | --- | --- | --- | --- |
| Markers of kidney damage (one or more) | | | Albuminuria (AER ≥ 30 mg/24 hours; ACR ≥ 30 mg/g [≥3 mg/mmol])  Urine sediment abnormalities  Electrolyte and other abnormalities due to tubular disorders  Abnormalities detected by histology  Structural abnormalities detected by imaging  History of kidney transplantation | | | | |
| Decreased GFR | | | GFR < 60 ml/min/1.73 m2 (GFR categories G3a–G5) | | | | |
| **GFR categories in CKD** | | | | | | | |
| **GFR category** | | **GFR (ml/min/1.73 m2)** | | | | **Terms** | |
| G1 | | ≥ 90 | | | | Normal or high | |
| G2 | | 60-89 | | | | Mildly decreased * | |
| G3a | | 45-59 | | | | Mildly to moderately decreased | |
| G3b | | 30-44 | | | | Moderately to severely decreased | |
| G4 | | 15-29 | | | | Severely decreased | |
| G5 | | < 15 | | | | Kidney failure | |
| * Relative to young adult level  In the absence of evidence of kidney damage, neither GFR category G1 nor G2 fulfill the criteria for CKD. | | | | | | | |
| **Albuminuria categories in CKD** | | | | | | | |
|  | **AER** | | | **ACR (approximate equivalent)** | | |  |
| **Category** | **(mg/24 hours)** | | | **(mg/mmol)** | **(mg/g)** | | **Terms** |
| A1 | < 30 | | | < 3 | < 30 | | Normal to mildly increased |
| A2 | 30-300 | | | 3-30 | 30-300 | | Moderately increased * |
| A3 | > 300 | | | > 30 | > 300 | | Severely increased ** |
| AER: albumin excretion rate, ACR: albumin-to-creatinine ratio, CKD: chronic kidney disease. | | | | | | | |
| * Relative to young adult level | | | | | | | |
| * * Including nephrotic syndrome (AER>2200 mg/24 hours (ACR>2200mg/g; >220mg/mmol)) | | | | | | | |

# Reference

1. Chapter 1: Definition and classification of CKD. *Kidney international supplements,* 3**:** 19-62, 2013
