# Supplementary material for: Serum calcification propensity is independently associated with disease activity in systemic lupus erythematosus
Source: PLoS One. 2018 Jan 24;13(1):e0188695. doi: 10.1371/journal.pone.0188695 (PMC5783342; doi:10.1371/journal.pone.0188695)
Supplement: S1 Fig — (DOC) [file pone.0188695.s007.doc]

**
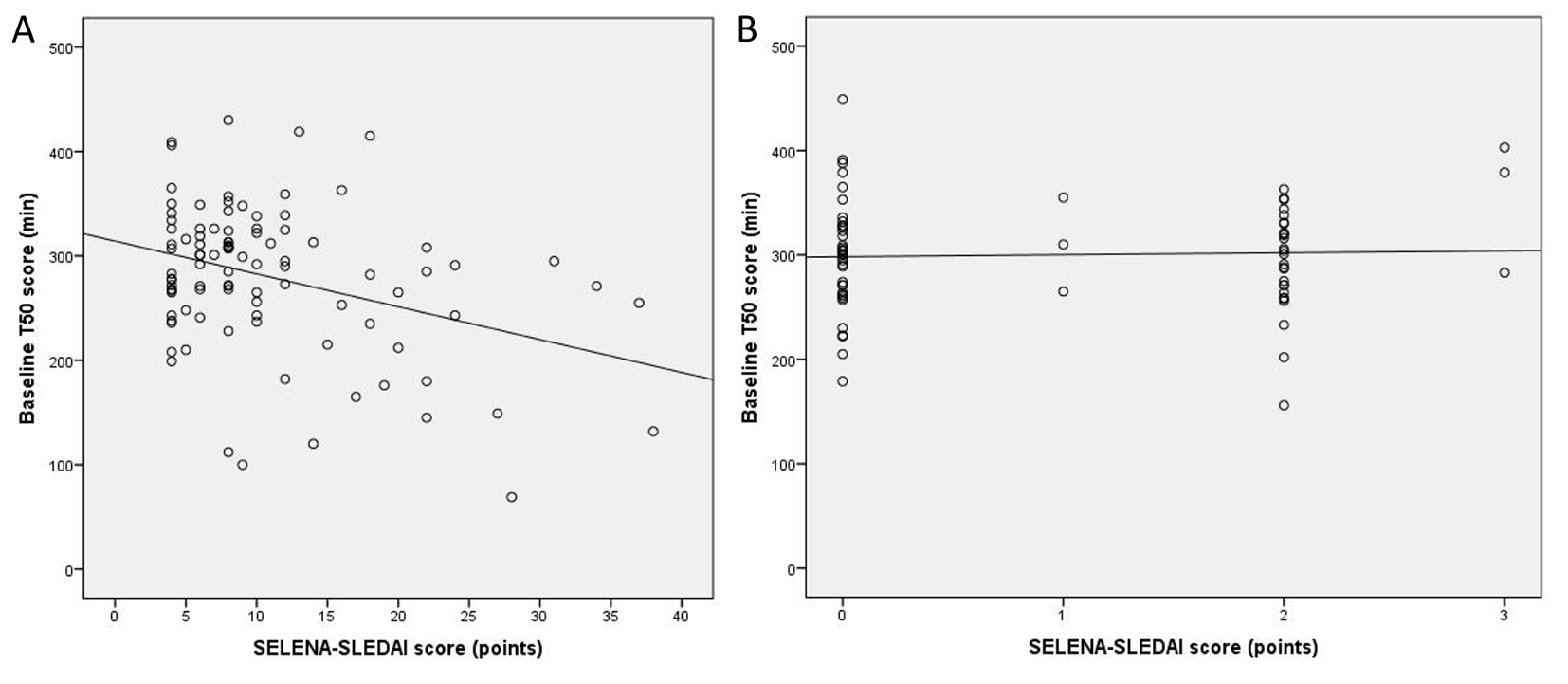
**

**S1 Fig 1.** Association between T50 and SELENA-SLEDAI score at baseline in disease activity. A. Association between T50 and SELENA-SLEDAI score at baseline in active disease (SELENA-SLEDAI score ≥4). B. Association between T50 and SELENA-SLEDAI score at baseline in inactive disease (SELENA-SLEDAI score <4)
